# The Impact of Regeneration and Climate Adaptations of Urban Green–Blue Assets on All-Cause Mortality: A 17-Year Longitudinal Study

**DOI:** 10.3390/ijerph17124577

**Published:** 2020-06-25

**Authors:** Zoë Tieges, Duncan McGregor, Michail Georgiou, Niamh Smith, Josie Saunders, Richard Millar, Gordon Morison, Sebastien Chastin

**Affiliations:** 1 School of Health and Life Sciences, Glasgow Caledonian University, 70 Cowcaddens Road, Glasgow G4 0BA, UK; Duncan.McGregor@gcu.ac.uk (D.M.); Michail.Georgiou@gcu.ac.uk (M.G.); Niamh.Smith@gcu.ac.uk (N.S.); Sebastien.Chastin@gcu.ac.uk (S.C.); 2 Biomathematics and Statistics Scotland, JCMB, The King’s Buildings, Peter Guthrie Tait Road, Edinburgh EH9 3FD, UK; 3 Scottish Canals, Canal House, 1 Applecross Street, Glasgow G4 9SP, UK; Josie.Saunders@scottishcanals.co.uk (J.S.); richard.millar@scottishcanals.co.uk (R.M.); 4 School of Computing, Engineering and Built Environment, Glasgow Caledonian University, 70 Cowcaddens Road, Glasgow G4 0BA, UK; Gordon.Morison@gcu.ac.uk; 5 Department of Movement and Sport Sciences, Ghent University, Watersportlaan 2, 9000 Ghent, Belgium

**Keywords:** green space, blue space, health, mortality, epidemiology, exposure, outdoor, GIS

## Abstract

Urban waterways are underutilised assets, which can provide benefits ranging from climate-change mitigation and adaptation (e.g., reducing flood risks) to promoting health and well-being in urban settings. Indeed, urban waterways provide green and blue spaces, which have increasingly been associated with health benefits. The present observational study used a unique 17-year longitudinal natural experiment of canal regeneration from complete closure and dereliction in North Glasgow in Scotland, U.K. to explore the impact of green and blue canal assets on all-cause mortality as a widely used indicator of general health and health inequalities. Official data on deaths and socioeconomic deprivation for small areas (data zones) for the period 2001–2017 were analysed. Distances between data zone population-weighted centroids to the canal were calculated to create three 500 m distance buffers. Spatiotemporal associations between proximity to the canal and mortality were estimated using linear mixed models, unadjusted and adjusted for small-area measures of deprivation. The results showed an overall decrease in mortality over time (β = −0.032, 95% confidence interval (CI) [−0.046, −0.017]) with a closing of the gap in mortality between less and more affluent areas. The annual rate of decrease in mortality rates was largest in the 0–500 m buffer zone closest to the canal (−3.12%, 95% CI [−4.50, −1.73]), with smaller decreases found in buffer zones further removed from the canal (500–1000 m: −3.01%, 95% CI [−6.52, 0.62]), and 1000–1500 m: −1.23%, 95% CI [−5.01, 2.71]). A similar pattern of results was found following adjustment for deprivation. The findings support the notion that regeneration of disused blue and green assets and climate adaptions can have a positive impact on health and health inequalities. Future studies are now needed using larger samples of individual-level data, including environmental, socioeconomic, and health variables to ascertain which specific elements of regeneration are the most effective in promoting health and health equity.

## 1. Introduction

Today, 55% of the global population lives in urban areas, a proportion that is expected to increase to 68% by 2050 [1]. Waterways are part of the urban fabric of most cities, with around 50% of people globally living within 3 km of urban inland waterways [2]. This rapid urbanisation, coupled with climate change, poses pressing new challenges for cities, such as increased pluvial and fluvial flood risk and reduced water quality caused by point source pollution and altered flow regimes [3]. Urban waterways have an important role to play in adapting to, and mitigating, climate change in urban environments, through reducing flood risks, alleviating the heat island effect (i.e., higher temperatures experienced in urban areas compared to nearby rural areas) and air pollution, reducing urban water pollution, and preserving biodiversity [4,5]. Such benefits can have wider socioeconomic consequences, creating attractive environments that encourage recreation and physical activity, fostering social interactions, and stimulating business investment and tourism. Importantly, urban waterways provide blue spaces (i.e., outdoor environments that prominently feature water) and green spaces (trees, grass, parks, etc.), which have been increasingly associated with benefits to health and well-being [6]. While urban parks and green spaces positively impact the health of urban dwellers, this tends to benefit only the most affluent urban populations [7]. Compared to parks and green urban spaces, urban waterways are often more equally distributed geographically and socioeconomically, and therefore have the potential to play a similar, if not more potent, role in improving urban health and reducing health inequalities. Indeed, recent research showed that blue spaces are most often visited by less affluent populations [8].

The evidence on health benefits of the natural environment has been mostly focused on green spaces [9,10]. However, in recent years, there has been a growing interest in the potential health-promoting effects of blue spaces (waters, rivers, etc.). There is relatively little literature on quantifying the health impact of blue spaces that suggests a positive association between greater exposure to outdoor blue spaces and improved physical and mental health and well-being [6]. However, few studies considered urban settings and most studies were cross-sectional in nature, which precludes making causal inferences. Thus, there is a lack of scientific evidence on, or practical understanding of, the potential health benefits of urban green and blue spaces and how this can be generated as a co-benefit of climate adaptation measures.

Recognising that urban waterways are underutilised assets with potential impacts on climate adaptation as well as public health, social cohesion, and recreation, European policies have called for the rejuvenation, repurposing, and development of the large and dense network of urban waterways in Europe [11]. In the post-industrial U.K., canals were left to dereliction in a spiral of environmental degradation and social exclusion. Over the past two decades, an ambitious programme of regeneration of derelict and disused canal assets has taken place in Scotland with the aim of reopening and revitalising the Forth and Clyde and Union Canals, reopening the waterways between the West and East coasts of Scotland to be fully navigable [12]. An important driver of the regeneration in North Glasgow was to provide climate adaptation for urban growth and use the canals as a sustainable urban drainage system [13]. The “Glasgow Smart Canal” project and so-called “Millennium link”, which is the largest canal restoration project in the U.K. to date, provides a unique opportunity to evaluate the effects of small-scale changes in urban blue and green spaces as a natural experiment.

To our knowledge, no study has yet explored the longitudinal impact of urban waterway regeneration on health outcomes. To address this research gap, the present study aimed to assess the quantitative effects of proximity to the canals on all-cause mortality in North Glasgow at the neighbourhood level over the 17-year period of canal regeneration. All-cause mortality represents a key health status indicator, which is widely used by policy makers and census (e.g., [14]) and reflects the “upper limit” of the disease severity continuum and the final common outcome of many health and nutrition problems.

## 2. Materials and Methods 

### 2.1. Study Design and Setting

The canal regeneration in North Glasgow during the years 2001–2017 provides a unique natural experiment to investigate the relationship between proximity to the urban blue spaces and mortality rates. Glasgow is Scotland’s most populous city with a total population of 578,710, according to the 2001 U.K. Census [15]. It is located along the North and South banks of the River Clyde in West Central Scotland and covers a total area of 175 km^2^. Glasgow city contains areas with morbidity and mortality rates among the worst in Western Europe, even after accounting for high levels of socioeconomic deprivation (the so-called “Glasgow effect” [16]). Moreover, people living in high deprivation areas within Glasgow have a lower life expectancy and increased risk of poor health outcomes compared to those living in low deprivation areas, indicating clear geographical health inequalities within Glasgow [17].

**Figure 1:**
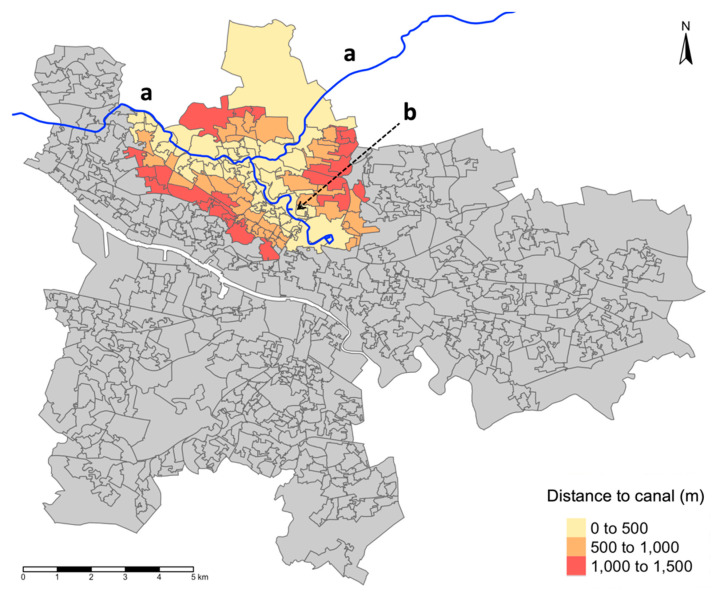
Glasgow city’s 694 data zones according to the 2001 Scotland Census and the Glasgow canal (blue line) comprising (**a**) the Forth and Clyde canal and (**b**) the Glasgow branch. The study area was split into three distance buffers (0–500, 500–1000, and 1000–1500 m), based on the Euclidian (i.e., straight-line) distance between the population-weighted centroids and nearest portion of the canal.

For the present paper, a study area was selected encompassing the canal network that runs through the North of Glasgow (area 33 km^2^) with a particular emphasis on the Glasgow Branch of the Forth and Clyde Canal where the majority of regeneration occurred (Figure 1). This area includes some of the most deprived communities of Scotland with particular pockets of deprivation in and around Maryhill and Possil, where the risk exposure to non-communicable diseases and poor mental health predominantly cluster [17]. Glasgow has large socioeconomic health inequalities, and the absolute gap in premature mortality between the most and least deprived has increased in recent years [18]. The upper and lower boundaries of the study area were the Glasgow city boundary (North) and the M8 motorway, which separates North Glasgow from the City Centre (South).

Because this research only used publicly available summary data at the neighbourhood-level, institutional review board approval was not required.

### 2.2. Data Zones

Aggregate-level census data on neighbourhoods (data zones) were obtained. Data zones were chosen as spatial units because they are the core small area geographical units used in Scotland, with a total of 6505 data zones created from the 2001 Census [19]. Data zones are groups of census output areas, with a population, on average, of between 500 and 1000 household residents. They nest within local authority boundaries (also known as council areas), and where possible, they have been constructed to respect physical boundaries and natural communities and reflect households with similar socioeconomic characteristics. Data zones were updated following the 2011 Census data. 

The study area in North Glasgow comprised 149 of the 694 Glasgow data zones based on the 2001 Census, and 157 of the 746 data zones based on the 2011 Census. All study data, including data zone boundaries and centroid shapefiles, were obtained from National Records of Scotland.

To enable time-series analysis on a consistent geographical basis, data zones linked to the 2001 and 2011 Census data were combined by freezing the geographical base (2001 data zones) and then mapping the 2011 data zones to these. A comparison between 2001 and 2011 data zones based on population and area (performed at the request of the Office of the Chief Statistician and Performance) was used to inform the data zone matching process [20]. One 2001 data zone was excluded because its population had become zero at the time of the 2011 Census. Where multiple 2011 data zones overlapped with a single 2001 data zone, the 2011 data zone with the greatest percentage fit in population size was selected [21]. Thirteen 2011 data zones matched to more than one 2001 data zone. These cases were inspected manually and were either replaced by the data zone that had the greatest degree of area overlap or were excluded, leaving a total of 145 data zones. It should be noted that there was no loss of overall geographical area during this matching process, but only a re-organisation of zones due to the change in the census process.

Next, distances between population-weighted centroids of the matched 2001 and 2011 data zones were computed, to check whether the matching yielded acceptable results to allow further analysis. The median distance between matched 2001 and 2011 data zone centroids was 49.2 m (interquartile range 12.73–13.83). The distances between 2001 and 2011 data zone centroids were less than 250 m in 139/145 of cases (93.8%). Only three pairs of matched data zones were between 500 m and 750 m apart. The results of the matching process were judged to be acceptable.

### 2.3. Exposure: Distance to the Glasgow Canal

The Euclidian distance, measured as the distance between two points in Euclidean space calculated using Pythagoras’ theorem (i.e., straight-line distance), between each 2001 data zone population-weighted centroid to the nearest portion of the Glasgow canal was calculated with the R package “sf” [22]. Data zones were then grouped according to this distance into 500 m buffer zones. This was informed by a systematic review on spatial dimensions used in studies on urban green–blue spaces and human health, which found that the median buffer distance used in physical health studies was 500 m (59 studies comprising a mixture of Euclidian and network buffer distances) [23]. The buffer zones of 0–500, 500–1000, and 1000–1500 m comprised 52, 36, and 26 data zones, respectively, and were included in the analysis. The number of data zones in more distant buffer zones was deemed too small (<20) to allow further analysis.

### 2.4. Outcome Variable: Mortality Rate

The annual number of all-cause registered deaths and mid-year population estimates for Glasgow city’s population were extracted for the period 2001–2017 from Scotland’s national dataset for deaths (freely available from https://statistics.gov.scot/). Mortality rates were expressed as a percentage of the data zone population ((number of deaths/population) × 100) to support presentation and interpretation of findings.

### 2.5. Covariates

The Scottish Index of Multiple Deprivation (SIMD) scores were used as indices of relative deprivation at the data zone level (available from https://statistics.gov.scot/). The SIMD is a publicly available continuous measure for identifying areas of deprivation across Scotland. The SIMD provides a comprehensive picture of material deprivation in small areas within Scotland. As such, the SIMD provides a consistent measure of health inequalities, and most measures used to assess inequality in Scotland are defined in terms of SIMD. Published SIMD scores represent a weighted sum of different domains: income; employment; housing; health; education; and geographic access. The additional domain, crime, was added in the SIMD 2012 but not included in the present analysis, because it was not available for the entire study period. In short, the income and employment domains are based on the number of people claiming relevant benefits, divided by either the total population (income) or the working-age population (employment) for each data zone. The housing domain is constructed by counting the number of people in households that are overcrowded or have no central heating, divided by the total household population. The health domain is based on various health indicators including emergency hospitalisations, mortality rates, low birth weight, etc. The education domain is based on different education indicators including school pupil absences, school leavers, pupil performance, and working-age people with no qualifications. Lastly, the access domain is based on drive time and/or public transport time to general practitioners, retail centres, petrol stations, schools, and post offices (details on the individual domains and weighting method are available from the Scottish Government website [24]). The full SIMD score was considered inappropriate to use in this study because the calculation of the SIMD health domain was based, in part, on standardised mortality rates, hence there would have been some circularity in investigating whether the full score predicted mortality (with possible over-adjustment of our analyses). Therefore, scores for the individual SIMD domains were used, excluding the health domain.

The weighted sum of the domain scores are ranked from most deprived to least deprived. For our study, SIMD is presented as population-weighted deciles from most deprived (1st decile) to least deprived (10th decile). SIMD decile scores were assigned according to the version of SIMD most relevant to the year in question: SIMD-2004 for years 2001–2003, SIMD-2006 for years 2004–2006, SIMD-2009 for years 2007–2009, SIMD-2012 for years 2010–2013, and SIMD-2016 for years 2014–2017 [25].

### 2.6. Statistical Analysis

Study variables were summarised as medians and interquartile ranges. The data were analysed using linear mixed models to examine the longitudinal impact of distance to the Glasgow canal and covariates on all-cause mortality rates. Mortality rates were log-transformed to enforce the constraint on no negative responses (i.e., the expected death rate must be positive) and to improve linearity and interpretability, after a small constant value (0.001) was added to enable transformation of zero values in the data. Back-transformed values for the change in mortality rates over time are reported to facilitate interpretation.

The main predictors were linear time (years 2001–2017) and distance to the canal (0–500 m (comparator), 500–1000, and 1000–1500 m) included as fixed factors in the model. Covariates were SIMD domains income; employment; housing, education, and geographic access. An unadjusted model and a model adjusted for SIMD domains were fitted to understand first the full impact of the regeneration including changes in housing, economy, and services, and second to explore the potential role of socioeconomic deprivation as confounder or effect modifier of the association between proximity to the canal and mortality. The models had a random intercept and random slope of time (equation provided in Supplementary File S1). Statistical significance was tested at *p* < 0.05, and 95% confidence intervals (CI) were reported. All analyses were conducted with R Version 3.6.1 [26]. Mixed models were fitted using the lmer function in the “lme4” package [27]. A supplemental linear mixed model analysis was conducted to compare the longitudinal changes in mortality rates between the 0–500 and 500–1000 m canal buffer zones with one of the most affluent areas in Glasgow (Hyndland and Dowanhill; median 2004 SIMD decile score 10, IQR = 9–10; Supplementary File S2).

## 3. Results

**Figure 2:**
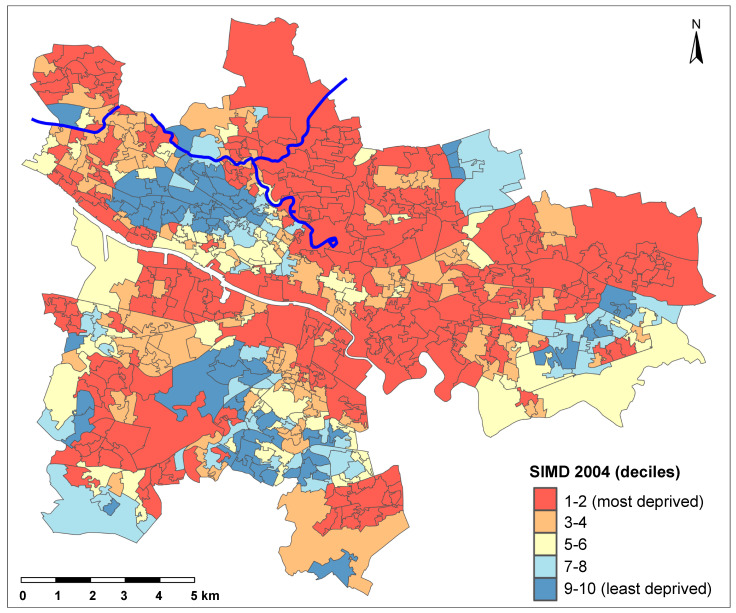
Glasgow city data zones by Scottish Index of Multiple Deprivation (SIMD) 2004 deciles, which relates to the years 2001–2003 (i.e., start of the study period). The blue line depicts the Glasgow canal.

The total population in the study area was 123,011 in 2001 which increased to 126,318 in 2017. Of these, the population living within 1500 m from the canal was 95,569 in 2001 and 95,493 in 2017. On the whole, a pattern of high deprivation levels according to SIMD decile scores was seen in the study area (Figure 2).

**Figure 3:**
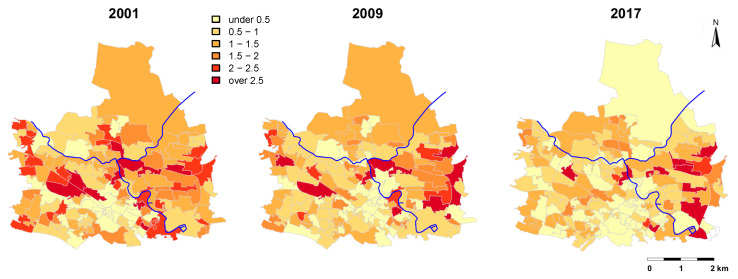
Mortality rates (%) in 2001, 2009, and 2017 in the study area of North Glasgow (see Supplementary Figure S1 for maps with mortality rates for each year during the study period). The blue line depicts the Glasgow canal. Data were obtained from National Records of Scotland.

**Figure 4:**
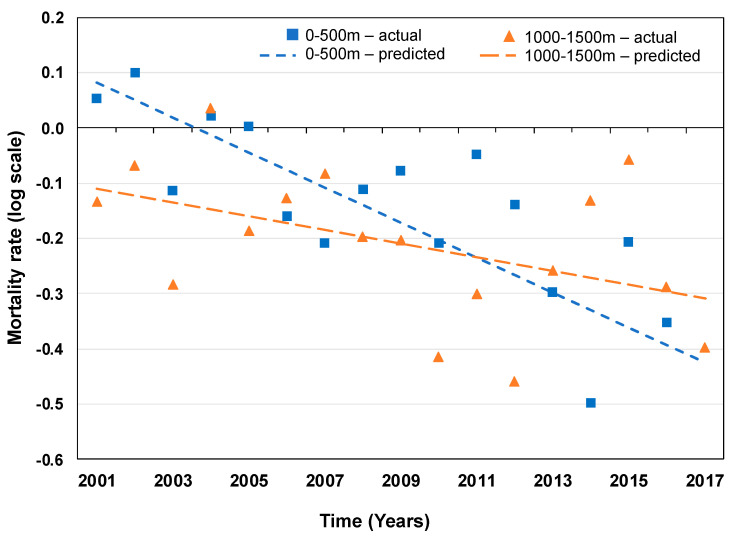
Mortality rates for the 0–500 and 1000–1500 m distance buffers around the canal in North Glasgow during the 2001–2017 time period of canal regeneration. The dashed lines represent the model-predicted trend in mortality rates for the 0–500 and 1000–1500 m distance buffers.

**Figure 5:**
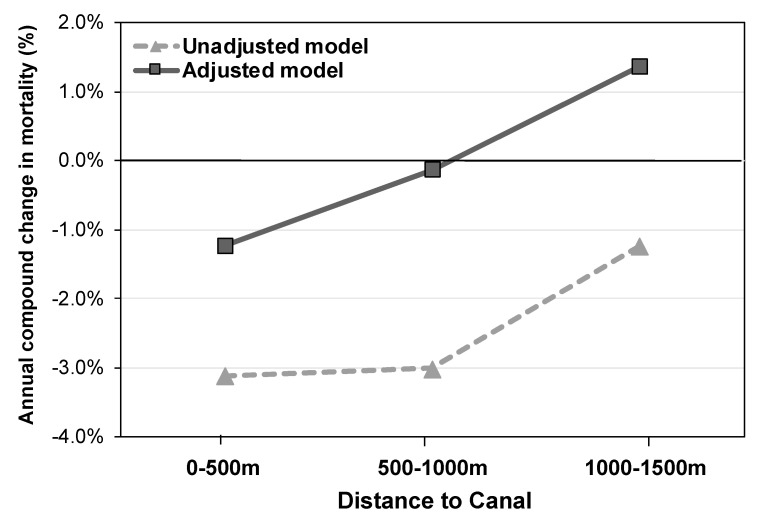
Annual compound change in mortality rate for three buffer zones (distance between 2001 data zone centroids and nearest portion of the canals), estimated from linear mixed models with and without adjustment for Scottish Multiple Deprivation Score (SIMD) decile scores for the domains income, housing, employment, education, and geographical access.

The overall decline in mortality rates apparent in Glasgow in the last two decades [28] was also evident in the canal region (β = −0.032, 95% CI [−0.046, −0.017], *p* < 0.001; Figure 3). Mortality rates in 2001 were 1.45%, 1.23%, and 1.21% for the 0–500, 500–1000, and 1000–1500 m buffer zones around the canal, respectively. In 2017, mortality rates in these buffer zones were reduced to 1.04%, 1.10%, and 1.06%, respectively. Thus, mortality rates were initially higher in the 0–500 m buffer zone compared to the 1000–1500 m buffer zone (gap in 2001 mortality rates =0.24), but this gap closed overtime to −0.02 in 2017 (indicating lower mortality rates close to the canal, Figure 4 and Figure 5).

The average rate of decline in mortality between 2001 and 2017 diminished with increased distance from the canal: the longitudinal decrease in mortality rates was largest in the 0–500 m buffer zone closest to the canal (−3.12% per annum, 95% CI [−4.50, −1.73]), with smaller decreases found in buffer zones further removed from the canal (500–1000 m: −3.01% per annum, 95% CI [−6.52, 0.62]; and 1000–1500 m: −1.23% per annum, 95% CI [−5.01, 2.71]; Figure 5). This corresponds to a model-predicted decrease in death rate over the 17-year study period of −0.43, −0.34, and −0.16 for the 0–500, 500–1000, and 1000–1500 m buffer zones, respectively.

The pattern of findings from the model adjusted for SIMD deprivation levels was similar to the results from the unadjusted model, such that the largest rate of decline in mortality was seen in the 0–500 m buffer zone (−1.23% per annum, 95% CI [−6.17, 3.80]), with a smaller rate of decline in the 500–1000 m buffer zone (−0.14% per annum, 95% CI [−7.17, 7.55] and even an increase in mortality (compound change of +1.37% per annum; 95% CI [−5.97, 9.68]) for the 1000–1500 m buffer zone (Figure 4 and Figure 5 and Supplementary Table S1; results from the supplemental analysis comparing the canal regions to the Hyndland and Dowanhill areas are presented in Supplementary File S2). This corresponds to a model-predicted change in death rates of −0.20, −0.03, and +0.23, respectively, for increasing buffer zones. Thus, the finding of larger rates of decline in mortality in regions closest to the canal (closing the gap with the average mortality rate in Glasgow as a whole) cannot be explained by changing deprivation levels alone.

## 4. Discussion

This observational study focused on an urban area undergoing substantial waterway regeneration as an empirical case to explore the association between blue and green urban space and mortality as an indicator of general health and health inequality. The study findings showed a faster decline in mortality rates in urban areas proximal to the canal compared to more distal areas over an interval that spanned nearly two decades. This association between a greater drop in annual mortality rates and proximity to the canal remained present after adjusting for regional deprivation, with a closing gap in mortality rate between the area closest to the canal and area further removed from the canal during the study period.

This natural experiment, therefore, suggests that the regeneration of blue and green space linked to urban waterways was associated with long-term reduction in mortality rates. The 3% mortality rate decrease corresponds to a substantial effect considering that mortality rate in Scotland’s poorest area is increasing by 1% per year since 2012 [29]. This highlights that substantial health benefits, relating to physical or mental illness and self-reported well-being, could be achieved from urban waterways regeneration synergistically with climate adaptation and economic regeneration. 

Our results suggest that the regeneration of the canals may have contributed to a change in the geographically unequal distribution of mortality rate and therefore towards greater health equality in the city. However, the findings should be interpreted with caution as the confidence intervals were wide and a direct causal link between the regeneration of specific canal assets (e.g., locks, bridges and waterway assets) and health benefits has yet to be established. Further, the associations between canals and mortality may be explained by residual confounding of sociodemographic factors that were not accounted for in the present study.

For the purposes of the present study, it was assumed that the canal regeneration occurred at a constant rate in time and space. An important next step is to link the timing and location of different types of assets (e.g., paths, bridges, water management, property, physical activity, and mobility infrastructure, etc.) to mortality and other health outcomes. This could reveal the impact on health (and health equity) of the regeneration, re-development, and repurposing of specific water infrastructure assets, which could, in turn, guide future development of inland waterways in Scotland and beyond. 

This study has several strengths. The present work is the first to leverage a successful regeneration programme of urban canals from closure to reopening and beyond as a natural experiment. Indeed, this is the first study to specifically address the relation between urban waterways and health during a major regeneration programme. The findings add to a small, but growing body of research evidence to support beneficial effects of urban blue (and green) spaces on health outcomes. The present study answered the need for longitudinal natural experiments to deepen our understanding of this field, as highlighted by Gascon and colleagues [6] in their recent review on blue spaces and health.

A number of limitations must be noted. First, the analysis did not account for sociodemographic variables including age and gender, although we checked trends in population shift in terms of age, gentrification, and improvement in socioeconomic variables. Further, there is no consensus on how to address the issue of administrative boundary changes following the 2011 Scotland Census (i.e., 2001 and 2011 data zones), to enable comparison of statistics over time. The present study adopted the “freezing” approach, which involved fixing the zone system according to the 2001 Census and recasting the 2011 Census boundary definition back in time. There are known disadvantages to this approach, including that the chosen zones become less appropriate to current applications over time [21]. This was a particular issue in the study area due to the scale of the regeneration and resulting changes in physical boundaries and population distribution over time. Therefore, a pragmatic but thorough matching process was conducted, using published matching information from a reliable source [20], complemented with manual inspection and calculation of distances between centroids to ensure that the matched zones did not deviate substantially.

Another study limitation is that SIMD indices were used as covariates to adjust for regional deprivation, a decision that was guided, in part, by data availability. However, capturing change over time in deprivation using SIMD is problematic because of changes to the methodology and some of the indicators used [25]. Further, straight-line distances were computed to estimate neighbourhood proximity to the canal, which could have resulted in overestimation of provision and accessibility of the canal and surrounding green space [30]. Future studies should consider using network distance buffers, which are constrained to a road, stream, or other linear transportation routes (i.e., real-world travel distance) [23,31].

Mortality or morbidity measures (e.g., cardiovascular disease, diabetes, stroke, etc.) may be an imperfect approximation of general health and health inequality [32]. However, mortality as an indicator of general health is widely used by policy makers and census (e.g., [14]). Mortality rate provides a general measure of the health of a population, which can signal a broad range of health problems. Moreover, differences in mortality rate are commonly used to assess the effect of exposure to natural environment on health inequalities (e.g., [33]). Future studies should consider using other health measures in addition to (or in lieu of) all-cause or cause-specific mortality, capturing both fatal and non-fatal health outcomes, to establish whether levels of health are improving meaningfully and inequalities are being reduced in connection to green and blue space environments. The focus should be on reducing the incidence and prevalence of conditions that cause ill health but not death and to reducing their impact on people’s lives. 

Benefits of green and blue spaces on health may derive from a variety of mechanisms, including the reduction of exposure to air pollutants [34], alleviation of the urban heat-island effect [35], and decreased land surface temperatures [36]. Further, proximity to green and blue spaces may benefit health through physical activity-related mechanisms [37], such as an increased rate of walking and cycling to work, generally increased moderate-to-vigorous physical activity, more dog walking, and greater street connectivity (e.g., [38,39]). Other possible causative mechanisms behind the green-blue space and health relationship are stress reduction and better mental health [40] (through increased social interaction, lower psychological distress, and a greater sense of community), self-perception of environmental restoration, and greater self-perceived evaluation of a neighbourhood [41].

While the census aggregation data used in the present study provide a valuable source of information due to their systematic collection, these data are known to involve a loss of information, which may have led to reduced ability to detect spatiotemporal patterns [42].

Future studies using individual-level data and a range of health variables are required to explore specific individual health benefits. Studies should consider environmental factors such as urban heat island and air quality as well as health and socioeconomic factors. Specifically, studies could investigate the role of individual (e.g., education and income) and neighbourhood (Index of Multiple Deprivation) socioeconomic status as effect modifiers of the association between proximity to blue spaces and health.

## 5. Conclusions

Our findings support the notion that regeneration of urban blue and green assets can have a strong positive impact on health and health inequalities in urban settings, although no conclusions about causality can be inferred, and confounding by other factors (e.g., environmental, socioeconomic) cannot be precluded. Future urban development should consider the impact on health of blue spaces in synergy with their use for economic development and climate adaptation. The health impact should be planned, monitored, and evaluated. Environmental and health factors should be studied together in order to obtain a more complete picture and allow for full cost–benefit analyses to inform policy makers usefully [43]. 

## Supplementary Materials

The following are available online at https://www.mdpi.com/1660-4601/17/12/4577/s1, File S1: Linear mixed model equation; Table S1: Linear mixed model results, without adjustments and adjusted for Scottish Multiple Deprivation Score (SIMD) decile scores for the domains income, housing, employment, education, and access; File S2: Results from supplemental linear mixed model analysis comparing longitudinal changes in mortality rates between canal buffer zones with an affluent comparator area in Glasgow (Dowanhill and Hyndland); Figure S1: Maps with mortality rates per year during the study period 2001–2017.
