# Supplementary material for: The Impact of Regeneration and Climate Adaptations of Urban Green–Blue Assets on All-Cause Mortality: A 17-Year Longitudinal Study
Source: Int J Environ Res Public Health. 2020 Jun 25;17(12):4577. doi: 10.3390/ijerph17124577 (PMC7344529; doi:10.3390/ijerph17124577)
Supplement: Supplementary file 1 [file ijerph-17-04577-s001.zip › Supplementary Figure S1.pdf]

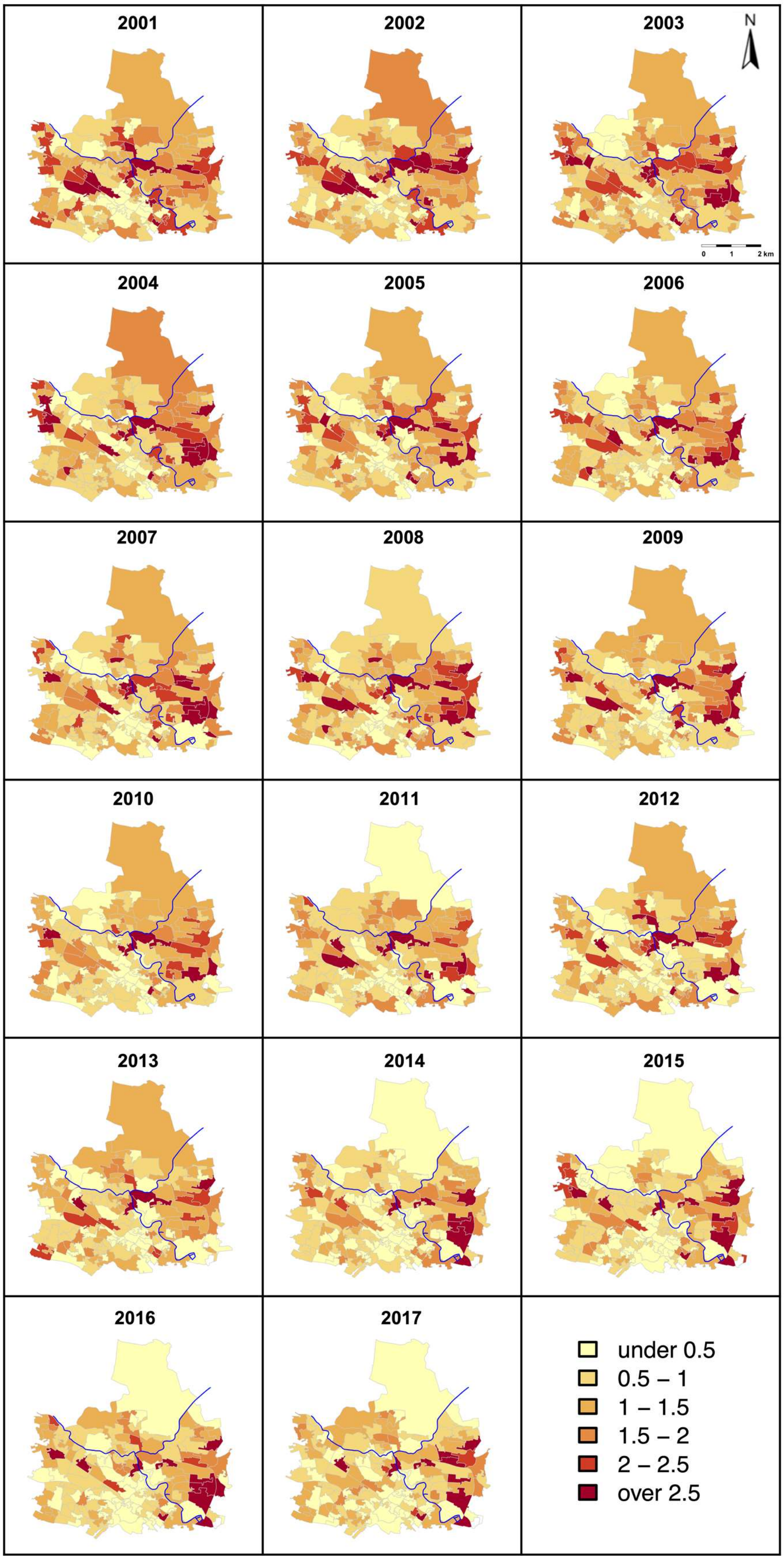

Supplementary Figure S1. Mortality rates (%) in the study area of North Glasgow for the study period 200-12017. The blue line depicts the Glasgow Canal. Data were obtained from National Records of Scotland.
