# Supplementary material for: The Impact of Regeneration and Climate Adaptations of Urban Green–Blue Assets on All-Cause Mortality: A 17-Year Longitudinal Study
Source: Int J Environ Res Public Health. 2020 Jun 25;17(12):4577. doi: 10.3390/ijerph17124577 (PMC7344529; doi:10.3390/ijerph17124577)
Supplement: Supplementary file 1 [file ijerph-17-04577-s001.zip › Supplementary File S1 FOR SUBMISSION.docx]

**Linear Mixed Model equation**

The equation for the adjusted mixed model for data zone (denoted by i) and time (denoted by j) is as follows:

| $Log\left( m_{ij} \right)=\left( \beta_{0}+\beta_{1}*d_{i}+\upsilon_{0i} \right)+\left( \beta_{2}+\beta_{3}*d_{i}+\upsilon_{1i} \right)*t+\beta_{4}*{SIMD}_{IN}+\beta_{5}*{SIMD}_{ED}+\beta_{6}*{SIMD}_{EM}+\beta_{7}*{SIMD}_{H}+\beta_{8}*{SIMD}_{AC}+\epsilon_{ij}$ | *(1)* |
| --- | --- |

where mortality is given by m_ij,_ distance by d, time by t, the SIMD domains Income, Education, Employment, Housing and Geographic Access defined by $\mathrm{SIMD}_{\mathrm{IN}}$, $\mathrm{SIMD}_{\mathrm{ED}}$, $\mathrm{SIMD}_{\mathrm{EM}}$. $\mathrm{SIMD}_{H}$ and $\mathrm{SIMD}_{\mathrm{AC}}$ respectively, and residual error defined as $\epsilon_{\mathrm{ij}}$. The model random effect terms $\upsilon_{i}$for individuals was assumed to be normally distributed with zero mean and variance $\sigma^{2}$.

| $\left[ \frac{\upsilon_{0i}}{\upsilon_{1i}} \right]\sim N\left( \left[ \frac{0}{0} \right],\left[ \begin{matrix} \sigma_{0}^{2} & \sigma_{01} \\ \sigma_{10} & \sigma_{1}^{2} \end{matrix} \right] \right)$ | *(2)* |
| --- | --- |
