# Supplementary material for: The Impact of Regeneration and Climate Adaptations of Urban Green–Blue Assets on All-Cause Mortality: A 17-Year Longitudinal Study
Source: Int J Environ Res Public Health. 2020 Jun 25;17(12):4577. doi: 10.3390/ijerph17124577 (PMC7344529; doi:10.3390/ijerph17124577)
Supplement: Supplementary file 1 [file ijerph-17-04577-s001.zip › Supplementary File S1.pdf]

Supplementary File S1

Linear Mixed Model equation

The equation for the adjusted mixed model for data zone (denoted by i) and time (denoted by j) is as follows:

$$Log(m_{ij}) = (\beta_0 + \beta_1 * d_i + \upsilon_{0i}) + (\beta_2 + \beta_3 * d_i + \upsilon_{1i}) * t + \beta_4 * SIMD_{IN} + \beta_5 * SIMD_{ED} + \beta_6 * SIMD_{EM} + \beta_7 * SIMD_H + \beta_8 * SIMD_{AC} + \epsilon_{ij} \quad (1)$$

where mortality is given by $m_{ij,}$ distance by d, time by t, the SIMD domains Income, Education, Employment, Housing and Geographic Access defined by $\mathrm{SIMD_{IN}}$, $\mathrm{SIMD_{ED}}$, $\mathrm{SIMD_{EM}}$. $\mathrm{SIMD_{H}}$ and $\mathrm{SIMD_{AC}}$ respectively, and residual error defined as ϵ_{ij}. The model random effect terms υ_i for individuals was assumed to be normally distributed with zero mean and variance σ^2.

$$\left[\frac{\upsilon_{0i}}{\upsilon_{1i}}\right] \sim N\left(\left[\frac{0}{0}\right], \begin{bmatrix} \sigma_0^2 & \sigma_{01} \\ \sigma_{10} & \sigma_1^2 \end{bmatrix}\right) \quad (2)$$
