# Supplementary material for: The Impact of Regeneration and Climate Adaptations of Urban Green–Blue Assets on All-Cause Mortality: A 17-Year Longitudinal Study
Source: Int J Environ Res Public Health. 2020 Jun 25;17(12):4577. doi: 10.3390/ijerph17124577 (PMC7344529; doi:10.3390/ijerph17124577)
Supplement: Supplementary file 1 [file ijerph-17-04577-s001.zip › Supplementary File S2 24 June.docx]

**Results from supplementary Linear Mixed Model analysis of the mortality rates in the 0-500m and 500-1000m areas around the canal in North Glasgow, compared with the areas of Dowanhill and Hyndland in the West End of Glasgow.**

We conducted a supplementary linear mixed model analysis with the main predictors linear time (years 2001-2017) and Glasgow area (Hyndland and Dowanhill (comparator), and 0-500m and 500-1000m buffer zones around the Glasgow canal, respectively) included as fixed factors in the model. Covariates in the adjusted analysis were SIMD domains Income; Employment; Housing, Education; and Geographic Access.

The longitudinal decrease in mortality rates in the comparator area of Hyndland and Dowanhill between 2001 and 2017 was -2.14% per year, 95% CI [-5.44%, 1.28%]. By comparison, the decreases in mortality rate in the 0-500 m and 500-1000m buffer zones around the canal were larger (0-500m: -3.12% per annum, 95% CI [-9.84%, 4.10%]; 500-1000m: -2.93% per annum, 95% CI [-9.80%, 4.46%]).

The model adjusted for SIMD deprivation levels suggested a longitudinal *in*crease in mortality rates in the comparator area of 3.77% per annum, 95% CI [-2.68%, 12.18%]. By comparison, a decrease in mortality rates was found in the 0-500 m and 500-1000 m buffer zones around the canal (0-500m: -1.15% per annum, 95% CI [-13.25%, 12.57%]; 500-1000m: -0.45% [95% CI -11.60%, 14.23%]).

The number of data zones in the comparator area was small (10 data zones). Note also the wide confidence intervals.

| Area | Compound change in mortality rate per annum | 95% Confidence Interval |
| --- | --- | --- |
| *Unadjusted analysis* |  |  |
| Comparator (Dowanhill and Hyndland) | 2.14% decrease p/a | -5.44%, 1.28% |
| 0-500m buffer around canal | 3.12% decrease p/a | -9.84%, 4.10% |
| 500-1000 buffer around canal | 2.93% decrease p/a | -9.80%, 4.46% |
| *Adjusted analysis* |  |  |
| Comparator (Dowanhill and Hyndland) | 3.7*7*% *in*crease p/a | -2.68%, 12.18% |
| 0-500m buffer around canal | 1.15% decrease p/a | -13.25%, 12.57% |
| 500-1000 buffer around canal | 0.45% decrease p/a | -11.60%, 14.23% |

Table. Linear mixed model results: Mortality rates in the 0-500m and 500-1000m areas around the canal in North Glasgow, compared with the areas of Dowanhill and Hyndland in the West End of Glasgow.


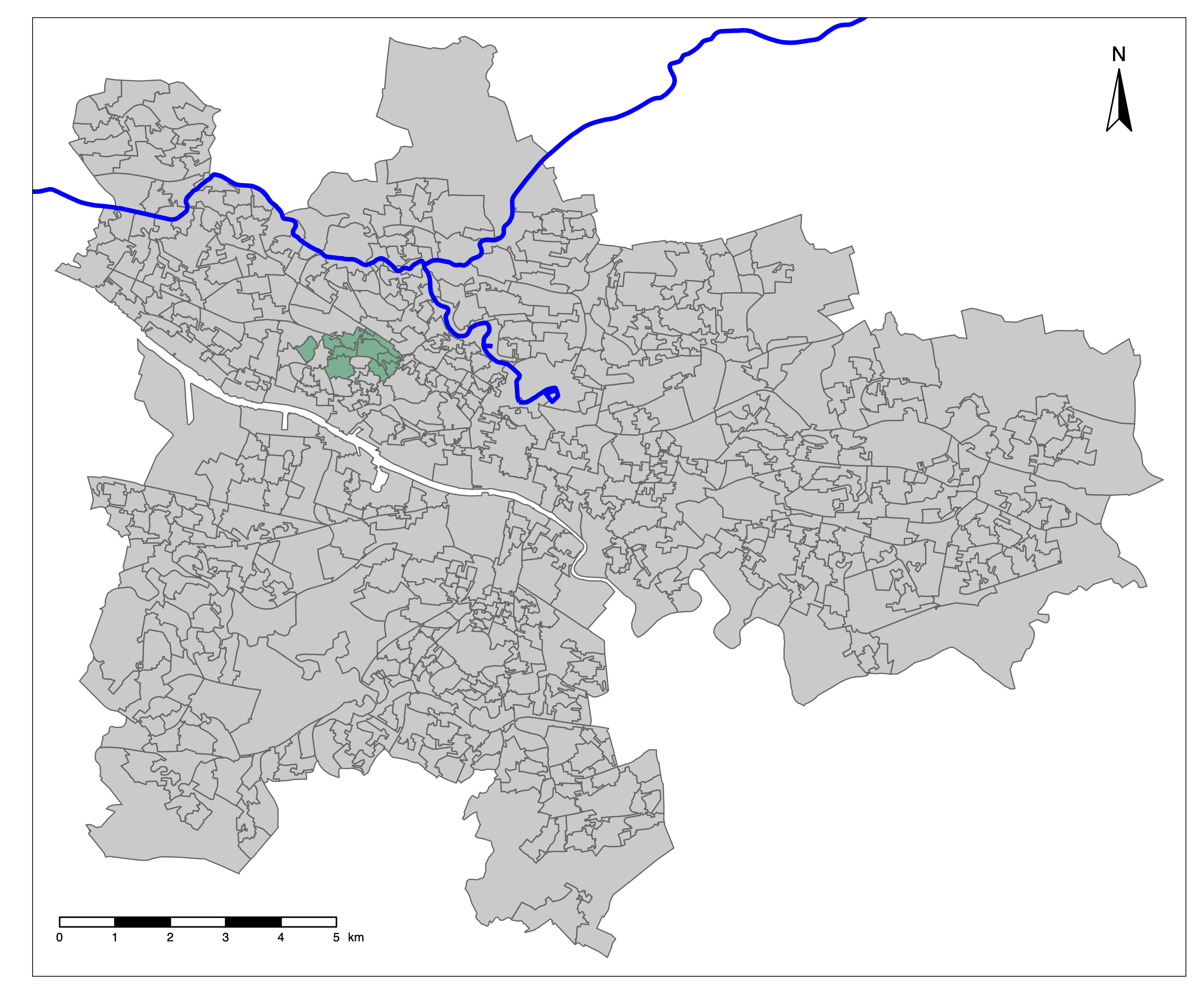


Figure. Location of comparator area in the West End of Glasgow (green), comprising Dowanhill and Hyndland. The blue line depicts the Lowlands canal.
