# Supplementary material for: The Impact of Regeneration and Climate Adaptations of Urban Green–Blue Assets on All-Cause Mortality: A 17-Year Longitudinal Study
Source: Int J Environ Res Public Health. 2020 Jun 25;17(12):4577. doi: 10.3390/ijerph17124577 (PMC7344529; doi:10.3390/ijerph17124577)
Supplement: Supplementary file 1 [file ijerph-17-04577-s001.zip › Supplementary Table S1 24 June.docx]

Table S1**.** Linear mixed model results, without adjustments and adjusted for Scottish Multiple Deprivation Score (SIMD) decile scores for the domains Income, Housing, Employment, Education and Access.

| **Effect** | **Estimate** | **95% CI** | **p-value** |
| --- | --- | --- | --- |
|  |  |  |  |
| **Unadjusted model** |  |  |  |
| (Intercept) | 0.114 | -0.145, 0.373 | 0.392 |
| year | -0.032 | -0.046, -0.017 | 0.000 |
| distance: 500-1000m | -0.198 | -0.603, 0.206 | 0.341 |
| distance: 1000-1500m | -0.213 | -0.660, 0.235 | 0.356 |
| year:distance (500-1000m) | 0.001 | -0.021, 0.024 | 0.923 |
| year:distance (1000-1500m) | 0.019 | -0.005, 0.044 | 0.130 |
|  |  |  |  |
| **Adjusted model** |  |  |  |
| (Intercept) | 0.197 | -0.556, 0.771 | 0.758 |
| year | -0.012 | -0.064, 0.037 | 0.654 |
| distance: 500-1000m | -0.094 | -0.420, 0.213 | 0.600 |
| distance: 1000-1500m | 0.006 | -0.385, 0.334 | 0.975 |
| year:distance (500-1000m) | -0.010 | -0.011, 0.035 | 0.423 |
| year:distance (1000-1500m) | 0.026 | 0.002, 0.055 | 0.106 |

Fixed effect: distance from 2001 data zone centroid to nearest portion of the canal.

Outcome measure: mortality rate ((N(deaths)/population)*100).
