# Supplementary material for: The Impact of Regeneration and Climate Adaptations of Urban Green–Blue Assets on All-Cause Mortality: A 17-Year Longitudinal Study
Source: Int J Environ Res Public Health. 2020 Jun 25;17(12):4577. doi: 10.3390/ijerph17124577 (PMC7344529; doi:10.3390/ijerph17124577)
Supplement: Supplementary file 1 [file ijerph-17-04577-s001.zip › Supplementary Table S1.pdf]

Table S1**.** Linear mixed model results, without adjustments and adjusted for Scottish Multiple Deprivation Score (SIMD) decile scores for the domains Income, Housing, Employment, Education and Access.

**Effect**	**Estimate**	**95% CI**	***p*-Value**
**Unadjusted model**			
(Intercept)	0.114	-0.145, 0.373	0.392
year	-0.032	-0.046, -0.017	0.000
distance: 500-1000m	-0.198	-0.603, 0.206	0.341
distance: 1000-1500m	-0.213	-0.660, 0.235	0.356
year:distance (500-1000m)	0.001	-0.021, 0.024	0.923
year:distance (1000-1500m)	0.019	-0.005, 0.044	0.130
**Adjusted model**			
(Intercept)	0.197	-0.556, 0.771	0.758
year	-0.012	-0.064, 0.037	0.654
distance: 500-1000m	-0.094	-0.420, 0.213	0.600
distance: 1000-1500m	0.006	-0.385, 0.334	0.975
year:distance (500-1000m)	-0.010	-0.011, 0.035	0.423
year:distance (1000-1500m)	0.026	0.002, 0.055	0.106

Fixed effect: distance from 2001 data zone centroid to nearest portion of the canal.

Outcome measure: mortality rate ((N(deaths)/population)*100).
